# Optimizing Diagnosis and Management of Ventilator-Associated Pneumonia: A Systematic Evaluation of Biofilm Detection Methods and Bacterial Colonization on Endotracheal Tubes

**DOI:** 10.3390/microorganisms12101966

**Published:** 2024-09-28

**Authors:** Ioana Roxana Codru, Bogdan Ioan Vintilă, Mihai Sava, Alina Simona Bereanu, Sandra Ioana Neamțu, Raluca Maria Bădilă, Victoria Bîrluțiu

**Affiliations:** 1 Faculty of Medicine, Lucian Blaga University of Sibiu,550169 Sibiu, Romania; ioanaroxana.bera@ulbsibiu.ro (I.R.C.); alina.bereanu@ulbsibiu.ro (A.S.B.); victoria.birlutiu@ulbsibiu.ro (V.B.); 2 County Clinical Emergency Hospital of Sibiu, 550245 Sibiu, Romania; sandraioana.neamtu@ulbsibiu.ro (S.I.N.); ralucamaria.vingerzan@ulbsibiu.ro (R.M.B.)

**Keywords:** biofilm, intensive care, ventilator-associated pneumonia (VAP), endotracheal tube (ETT)

## Abstract

Healthcare-associated infections, such as ventilator-associated pneumonia and biofilm formation on intubation cannulas, impose significant burdens on hospitals, affecting staffing, finances, and patient wellbeing, while also increasing the risk of patient mortality. We propose a research study aimed at exploring various methodologies for detecting these infections, discovered in the biofilm on medical devices, particularly tracheal cannulas, and understanding the role of each method in comprehending these infections from an etiological perspective. Our investigation also involves an analysis of the types of endotracheal tubes utilized in each case, the bacteria species identified, and strategies for combating biofilm-associated infections. The potential impact of our research is the substantial improvement of patient care through enhanced diagnosis and management of these infections.

## 1. Introduction

Healthcare-associated infections, also known as nosocomial infections, are a significant concern in hospital settings. These infections can lead to increased morbidity and mortality rates among patients. It is crucial for healthcare facilities to implement strict infection control measures to mitigate the risk of healthcare-associated infections and ensure the safety of all patients under their care. Among these infections, those related to antibiotic use, *Clostridioides difficile* enterocolitis [1,2], and ESKAPE group bacteria infections in intensive care unit patients [3], stand out.

Ventilator-associated pneumonia (VAP) is a common infection in the ICU, with reported incidences varying from 5% to 40% depending on the specific setting and diagnostic criteria. The correlation between ventilator-associated pneumonia (VAP), extended mechanical ventilation, and ICU stays is well established. The estimated mortality rate attributable to VAP is particularly high, exceeding 10%, especially in surgical ICU patients and those with moderate severity scores upon admission [4].

Despite recent advancements in microbiological tools, diagnostic criteria for VAP remain debatable, making it challenging to create an appropriate treatment or prevention plan [4]. Biofilms on endotracheal tubes can complicate VAP diagnosis by being difficult to detect, and they may contribute to false negative results in traditional culture methods. Distinguishing harmless colonization from actual infection is a significant challenge in VAP diagnosis. Current detection methods may have limitations in identifying biofilms associated with VAP, and the variability in bacterial colonization makes standardized diagnosis difficult. There is a need for more accurate or comprehensive diagnostic approaches to address this challenge and understand the relationship between biofilm formation, bacterial colonization, and the development of VAP [5,6].

VAP also imposes a substantial economic burden. In a recent assessment, the attributable median cost of VAP per patient stay in the USA was estimated to be $40,144 [7,8].

Endotracheal tubes (ETTs), essential devices used in mechanical ventilation for critically ill patients, are particularly susceptible to biofilm colonization due to their prolonged contact with the patient’s airways and exposure to diverse microbiota [9].

Biofilms are complex, dynamic communities of microorganisms that adhere to surfaces and are encased in a self-produced extracellular matrix [10,11]. In healthcare settings, biofilm formation on medical devices poses a significant challenge to patient care and safety [12]. Biofilm growth on endotracheal tubes significantly complicates the treatment of ventilator-associated pneumonia (VAP). These communities of microorganisms create a protective environment that increases antibiotic resistance up to 1000-fold compared to free-floating bacteria. They also serve as a persistent source of infection, interfering with host immune responses and creating physical barriers that impede antibiotic penetration. Additionally, bacteria within biofilms can enter slow-growing states less susceptible to antibiotics and communicate through quorum sensing, potentially increasing virulence. These factors collectively make VAP treatment more challenging, often requiring higher antibiotic doses, longer treatment durations, or even mechanical interventions such as tube replacement [11].

The biofilm formation process on ETTs typically begins within hours of intubation, as planktonic bacteria adhere to the tube’s surface and initiate the production of extracellular polymeric substances (EPS). This EPS matrix provides structural integrity to the biofilm and protects the microorganisms from host immune responses and antimicrobial agents. Over time, the biofilm matures, developing into a complex three-dimensional structure that can shelter a variety of bacterial species, including potential virulent pathogens [11,13].

Several methods have been developed to identify bacterial burden, structure, and architecture in biofilms, each with its own specificities. Sonication is a novel approach among these methods, although it is not a diagnostic method. Rather, it is used to enhance the specificity and sensitivity of methods used for etiologic diagnosis [14,15,16].

Methods for detecting biofilms on endotracheal tubes typically involve the use of microscopic techniques such as scanning electron microscopy, culture-based methods, and molecular approaches like PCR or fluorescence in situ hybridization. These methods play a crucial role in identifying and characterizing biofilms that may not be evident through standard clinical cultures. Accurate detection is essential for gaining a comprehensive understanding of biofilm formation, which directly influences the diagnosis and management of ventilator-associated pneumonia. Additionally, these methods are instrumental in assessing the efficacy of preventive measures and treatments designed to mitigate biofilm formation on medical devices [15].

Objectives: The microbial composition of biofilms, complex structures formed by microorganisms, is closely related to the development of ventilator-associated pneumonia [17]. The main objective of this review is to analyze various methods of identifying and describing biofilms on tracheal cannulas and assess the role of sonication among these methods. We also examine the existing knowledge gaps and pinpoint potential areas for future research. We explore the clinical impact of routine biofilm detection on VAP diagnosis, prevention, and treatment to improve early VAP detection and interventions.

## 2. Materials and Methods

This systematic review was conducted with meticulous attention to detail in accordance with the PRISMA (Preferred Reporting Items for Systematic Reviews and Meta-Analyses) guidelines. No formal protocol was registered before initiating the review, ensuring a comprehensive and unbiased approach.

The search included exploring three databases: PubMed, Scopus, and Google Scholar. We utilized the following keywords: sonication, biofilm, ventilator-associated pneumonia (VAP), endotracheal tube (ETT), and intensive care, using the Boolean operators ‘OR’ and ‘AND’. We also used a particular time interval for the articles chosen for the paper: 2019–2024.

After the initial search, we found 143 articles. After three rounds of excluding irrelevant articles, we were left with 39 studies. The articles were manually and automatically excluded through the Mendeley Desktop version, to where they were exported.

Eligibility criteria: Our study considered articles written in English within a specific time frame focusing on biofilms and ventilator-associated pneumonia. The included articles were required to meet the following criteria: (1) address bacterial pneumonia; (2) describe biofilms on solid or fluid surfaces related to the lungs; (3) include methods for biofilm description.

Exclusion criteria: We excluded case reports, case series, study protocols, and descriptive narratives. We also removed duplicate articles and those without full-text access. Furthermore, articles not describing biofilms, not involving endotracheal cannulas, and those not in English were also excluded.

**Figure 1:**
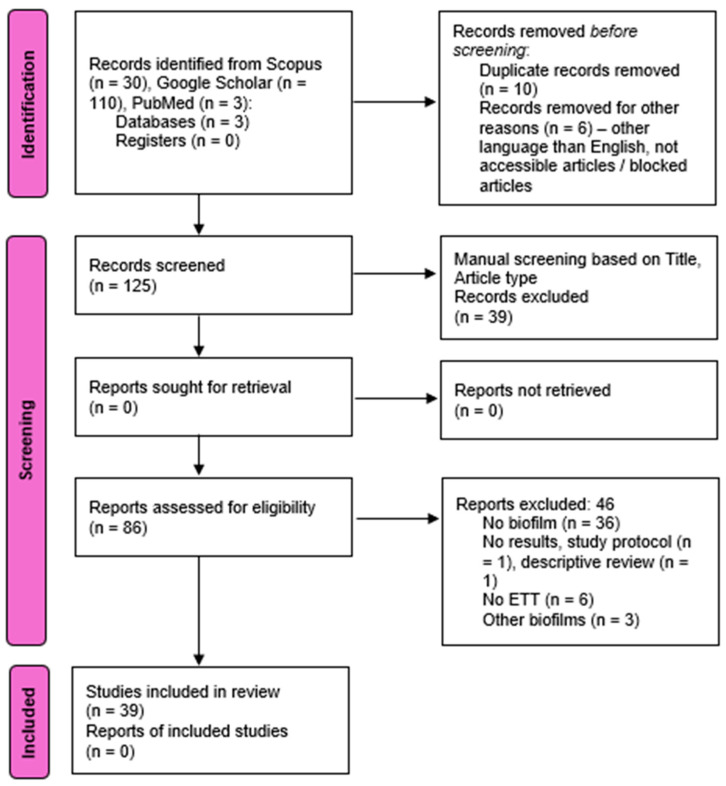
PRISMA flow diagram.

All articles were subject to bias assessment. Research on animal models is susceptible to bias, as the findings may not directly apply to human patients in the intensive care unit. The selection process is depicted in Figure 1, represented by the PRISMA chart.

## 3. Results

We have compiled the findings from the research we reviewed into Table 1.

In a comprehensive analysis, 39 studies were reviewed to collect data on the techniques utilized for detecting biofilms on tracheal cannulas, including endotracheal tubes and tracheostomy tubes, and to determine the potential impact of sonication on obtaining more detailed information. The analysis also involved evaluating the types of cannulas utilized, the specific bacteria under investigation, and whether the research was carried out in a clinical or laboratory environment.

**Figure 2:**
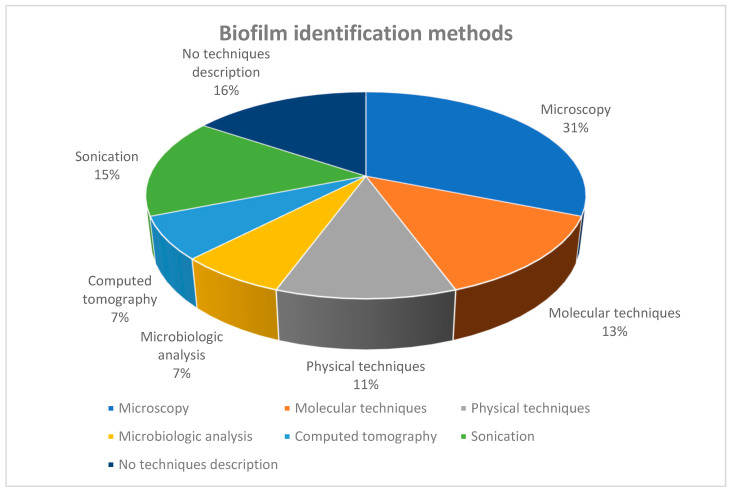
Methods used for identification of the biofilm formed on biotic and abiotic surfaces.

In the literature that we examined, microscopy was the most frequently used technique for biofilm identification, utilized in approximately one-third of the studies (31%) (Figure 2). However, it is essential to emphasize that microscopy encompasses a range of methods. It is not limited to simple optical microscopy employing various staining techniques. Instead, it includes several electron microscopy techniques utilized for identifying and characterizing biofilms and their components: confocal microscopy (3 studies) [40], scanning electron microscopy (7 studies) [7,14,20,25,28,35,37], and total internal reflection fluorescence microscopy (1 study) [23].

**Figure 3:**
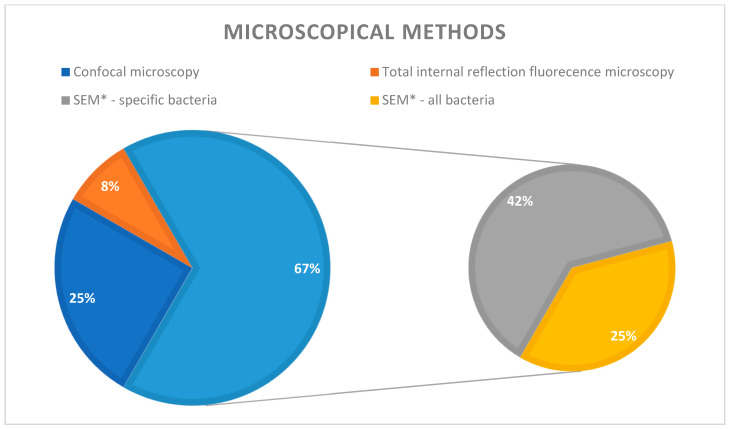
The use of microscopy in the studies analyzed (* SEM = scanning electron microscopy).

According to the studies’ analysis, scanning electron microscopy (SEM) emerges as the predominant method of investigation, being utilized approximately two-thirds of the time (69%). Within this, 42% of the instances involved the identification of specific bacteria strains that were the focus of the study (Figure 3).

Microscopy allowed the researchers to delineate biofilm components and discern various bacterial species. Some articles focused on a specific pathogen, while others described all bacteria present in the biofilm adhering to medical devices (Figure 2 and Figure 3).

In a lower percentage of the studies (7%), another visual method for biofilm identification was used: computed tomography (CT).

In the examined literature, a range of methods were applied to detect the bacteria responsible for biofilm formation on endotracheal tubes (ETTs). These methods included physical techniques (11%) such as spectrometry and flow cytometry, genetic approaches (13%) such as polymerase chain reaction and RNA-sequencing analysis, as well as traditional microbiological cultures in specific growth conditions (7%). The last two investigative methods can be utilized in clinical settings. In the current review, the utilization frequency was low, representing only 20% of all the studies. This low frequency could be attributed to the lack of novelty in these methods.

Some of the authors also used sonication as an investigative technique. Sonication alone, however, cannot offer any information on bacteria in the planktonic state or in biofilm.

In this review, 15% of the analyses utilized sonication to enhance the sensitivity of other biofilm identification methods. All the authors made use of sonication baths, with a median sound wave frequency of around 40 kHz and a power of approximately 0.22 W/cm^2^. These parameters have been demonstrated to be suitable for maintaining the viability of bacteria within biofilms.

In a study published in 2022, Cifuentes E.A. et al. highlighted the importance of sonication in advancing our understanding of biofilm composition [22]. They applied sonication to extract microorganisms from invasive medical devices, such as tracheal tubes, followed by DNA extraction using the Powersoil Kit. Their investigation’s results were notable, as they not only identified the common ESKAPE group bacteria but also discovered less prevalent species, including *Proteobacteria*, *Fusobacteria*, *Actinobacteria*, *Firmicutes*, and *Bacteroides*.

**Figure 4:**
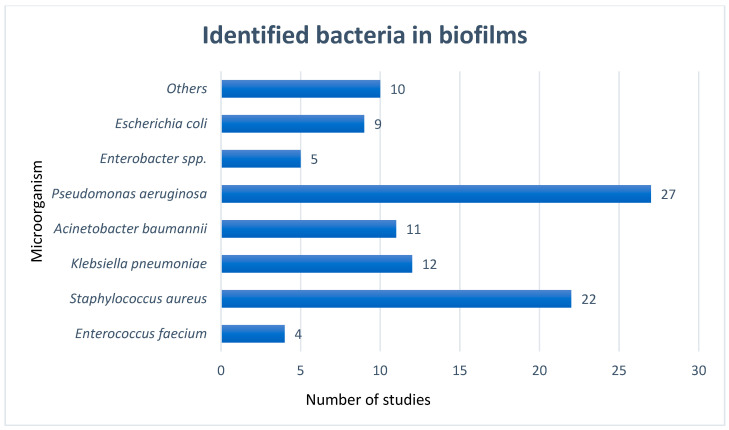
Bacteria that were identified in the biofilms formed on different invasive medical devices (mainly tracheal cannulas).

In Figure 4, we summarize the bacterial species identified in the studies. It should be noted that, while some researchers focused on specific types of species, others aimed to illustrate all microorganisms that could be part of bacterial communities.

The difference between the two main types of research lies in the types of tracheal tubes used when conducting the search. In most studies, mainly in clinical settings with human subjects, the tracheal tubes used were the more common PVC types, probably the most largely available kind of tubes (18 out of 39 studies—46.15%). Two studies compared the biofilm composition formed on simple, uncoated PVC tubes with that on coated ones. In a study conducted in 2021 by Lattore M.C. et al., Ceragenin CSA-131 was employed to inhibit biofilm formation. Ceragenins are synthetic amphipathic molecules derived from cholic acid, designed to replicate the activity of endogenous CAMPs (cationic antimicrobial peptides) [37]. These molecules are cost-effective to produce, non-toxic, and resistant to proteolysis [55,56,57]. Lattore demonstrated a decrease in biofilm formation on the surface of the coated tubes in the study, but the variance was not statistically significant [37].

In the subsequent study carried out by Roy S. et al. in 2022 [49], the focus was explicitly on *Acinetobacter baumannii*. This pathogen, a member of the ESKAPE group, has been frequently isolated from patients with late-onset VAP (up to one-third of all ICU cases as reported by Malacarne et al., even since the beginning of 2000 [58]). Roy described a series of naturally occurring antibiotics that use essential oils for tube coating to inhibit *Acinetobacter* biofilm formation. These substances appeared to have a positive impact, but the results remain inconclusive [49].

The authors researched various substances with theoretical antimicrobial activity in an additional 14 studies from the referenced list. The objective was to develop coatings for tracheal tubes that would prevent bacterial adherence and biofilm formation on their surfaces. For this purpose, noble metals, other chemical substances (chlorhexidine, methylene blue, ciprofloxacin), and even live coatings consisting of bacteriophages were used [5,9,20,23,32,37,38,43,44,50,51,53,54,59].

In the field of noble metal coatings, researchers used various metallic materials, or their oxides, inserted into or coated on polymeric surfaces. Two commonly used metals were silver and titanium. Given the increasing aggressiveness and virulence of *Klebsiella* spp., it is crucial to enhance biofilm prevention. Studies have shown that embedding TiO_2_ nanoparticles in the coatings of endotracheal tubes plays a significant role in preventing nosocomial infections caused by KPC-producing *Klebsiella pneumoniae*. However, it is worth noting that, despite their effectiveness, TiO_2_ nanoparticles may potentially contribute to certain types of cancer, as reported by the International Agency for Research on Cancer (Bereanu et al., 2024) [21]. On the other hand, silver has well-established antimicrobial properties. Since silver is already used in the general population, it is considered safe to use silver nanoparticles, as they do not exhibit toxic properties. Therefore, silver coatings can prevent surface colonization, even on tracheal tubes (Marcut L et al., 2023) [9]. In a study conducted in 2023, Lethonggam et al. demonstrated a significant reduction in *Pseudomonas aeruginosa* burden in the endotracheal tubes and lungs of pigs with VAP. Additionally, their study showed that the approach was safe when used on the porcine model [38].

In 2020 and 2021, Oliveira et al. studied the role of bacteriophages in preventing biofilm formation. They isolated phages from domestic sewage, which belonged to the order *Caudovirales* and *Myoviridae family*. They demonstrated the efficacy of these phages on almost 70% of *Pseudomonas aeruginosa* strains (69.7%), both in planktonic form and when embedded in bacterial biofilms. The phages showed good lytic activity on multidrug-resistant *Pseudomonas*. In a subsequent study, they monitored biofilm formation over a longer interval and once again demonstrated the important role of phages in reducing biofilm formation in endotracheal tubes. The authors suggested that this research paves the way for developing phage coatings and treatments to prevent nosocomial pulmonary infections with aggressive *Pseudomonas* strains [43].

**Figure 5:**
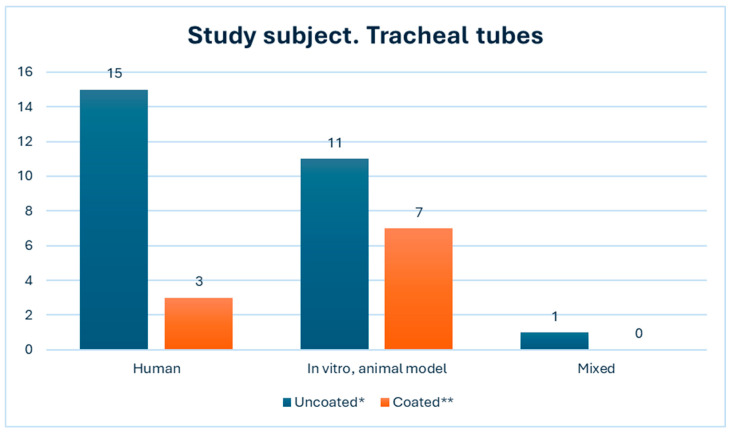
Tracheal tubes used in the studies. The subject of the study. * usual PVC tracheal tubes, ** coated tubes with noble metals, antibiotics, phages, and other.

Figure 5 represents the utilization of both coated and uncoated tracheal tubes in the respective studies, along with the classification of the study subjects: human, in vitro/animal model, or both. The graph visually represents an equal distribution of studies conducted on human populations and within laboratory settings. Furthermore, one study encompassed both clinical and laboratory models [42].

## 4. Discussion

This review, aiming to consolidate the research findings of various authors who have employed diverse methodologies to study biofilms, has considered a comprehensive range of study conditions and subjects, as well as preventive and treatment measures. The results of our investigation align with and support the findings reported by Costa-Orlandi et al. in 2017 [59]. Despite the latter’s focus on biofilms composed of fungi and yeasts, these add further credence to the burgeoning body of evidence in this field. This comprehensive approach ensures that all aspects of biofilm research have been considered and addressed.

Microscopy is the preferred method for direct visualization. Microscopic techniques have specific strengths and limitations that go beyond just identifying microorganisms. Most techniques allow for thorough analysis of biofilm formation through direct visualization and detection [60,61]. However, scanning electron microscopy can degrade the structure and morphology of the sample during preparation by fixation, dehydration, and staining [60,61]. On the other hand, electron microscopy has the advantage of preserving the biofilm architecture by using different sample preparation methods [59].

Confocal laser scanning microscopy comprehensively describes the biofilm’s architecture and composition, including the extracellular matrix (polysaccharides, proteins, nucleic acids, lipids). The microscope also reveals the spatial relationship between the biofilm and the surrounding structures [59]. In a study conducted in 2022, Maldiney T. et al. identified two distinct shapes of biofilms: mushroom-shaped and ribbon-shaped. Both types comprise similar bacteria, although certain species are characteristic of each shape. The mushroom-shaped biofilms are predominantly composed of *Staphylococcus aureus* and *Enterococcus cloacae*. Additionally, specific species found in this type of biofilm include *Enterococcus xiangfangensis, Streptococcus pneumoniae, Streptococcus oralis,* and *Hafnia alvei*. On the other hand, ribbon-shaped biofilms are primarily comprised of *Staphylococcus haemolyticus*, *Staphylococcus epidermidis*, *Escherichia Coli*, and *Enterococcus faecalis*, along with multiple species of *Enterobacter* [60]. It is important to emphasize that confocal microscopy provides a larger picture which can guide the researcher’s focus.

Electron, scanning electron, and confocal laser microscopy pose accessibility challenges at the point of care and require specialized personnel for probe handling and result interpretation.

Computed tomography (CT) is a promising visual technique for biofilm analysis. It generates 2D and 3D images, providing a comprehensive view of the biofilm’s composition, structure, and architecture. Technological advancements have improved image quality through enhanced signal intensity auto-scaling in imaging devices. However, the limited accessibility of this method, primarily due to its application in laboratory research and the need to collect biofilm specimens from potential patients, has hindered its widespread use [60,61].

While bacterial cultures are prevalent in hospital laboratories, they are time-consuming, typically taking several days to yield conclusive results. In contrast, genetic methods offer rapid identification of pathogens and insight into potential antibiotic resistance.

It is crucial to note that in 2022, Amar K.T. et al. used an RNA gene amplification technique followed by Sanger sequencing to identify a wide range of bacteria. They detected the usual pathogens responsible for nosocomial pneumonia, ESKAPE group bacteria, and other less common microorganisms in hospitals. These include *Streptococcus infantis*, *Gemella haemolysans*, *Meiothermus sylvanus*, *Shlegelella aquatica*, *Rothia mucilaginosa*, *Serratia nematodiphila*, and *Enterobacter asburiae*. Some of these microbes require special growth conditions, which may explain why standard microbiological tests fail to detect them. Interestingly, two of these bacteria, *Meiothermus sylvanus* and *Serratia nematodiphila*, are not typically associated with human infections and have not been previously described as human pathogens [19].

These techniques can be implemented at the point of care; however, it is noteworthy that not all intensive care units, especially in low-income countries, are equipped with the necessary apparatus for genetic bacterial identification.

Sonication is a process that makes use of sound energy to agitate particles or discontinuous fibers in a solution or to remove cells from certain surfaces [62,63,64,65]. This method employs acoustic energy or sound waves to transform an electrical signal into a physical vibration with a specific frequency and amplitude, which is then directed toward a substance [66,67,68]. Sonication, while not a diagnostic method on its own, is utilized to enhance the sensitivity of other techniques. By employing various protocols for dislodging biofilms using sound waves, the collected samples will contain more bacteria from the biofilm. Careful consideration should be given to choosing the protocol depending on the subsequent steps of the microbiological identification process. In this review, tracheal cannulas are the abiotic surfaces under investigation. In this context, the biofilm must be dislodged from the surface, sonicated, and subjected to another identification method. When genetic analysis is the sole method used, the microorganisms need not necessarily be alive, as this method solely detects genetic material. However, if the intention is to culture the samples in growth media, the bacterial material must be viable in order to grow and form colonies. Therefore, the sonication protocol, including wave frequency and power, must be selected meticulously. Along with previously discussed diagnostic methods, sonication can be combined with physical or microbiological techniques.

It should be emphasized that sonication should be used only to better evaluate the microorganisms on different biotic or abiotic surfaces.

Many authors have focused on bacteria in the ESKAPE group, which includes *Enterococcus faecium*, *Staphylococcus aureus*, *Klebsiella pneumoniae*, *Acinetobacter baumannii*, *Pseudomonas aeruginosa*, and *Enterobacter* spp. These six bacterial pathogens are highly virulent and are often associated with ventilator-associated pneumonia (VAP). In some cases, the group is expanded to include *Escherichia coli*. Some authors have found that, in addition to the bacteria commonly isolated in intensive care units, many other bacteria can be present in the biofilms adhering to medical devices.

It is imperative to note that investigations focusing on human subjects predominantly utilize standard polyvinyl chloride (PVC) endotracheal tubes, as the toxicological profile of alternative substances employed in antimicrobial coatings has yet to be fully elucidated. Two primary areas of concern warrant careful consideration: the potential local effects of these coatings on the tracheal mucosa and their systemic toxicity profile. Preliminary data from in vitro studies and animal models suggest promising results regarding the antimicrobial efficacy of these coatings against both planktonic bacteria and established biofilms. However, further research is necessary to bridge the translational gap between preclinical findings and clinical application.

The antimicrobial activity observed in controlled laboratory settings and animal studies provides a foundation for cautious optimism. Nevertheless, the complex interplay between coating materials, host physiology, and microbial ecology in the human respiratory tract necessitates rigorous clinical evaluation before widespread implementation can be considered. The potential benefits of reduced bacterial colonization must be carefully weighed against the risks of adverse tissue reactions or systemic effects, underscoring the need for comprehensive safety assessments in human subjects.

The outcomes of identifying biofilms can differ between in vitro (laboratory) and clinical settings due to various factors such as environmental complexity, host immune interactions, polymicrobial nature, and different growth conditions. In vitro studies use controlled environments, while clinical settings involve complex and dynamic conditions within the human body, including interactions with the immune system. These differences can significantly impact biofilm formation, structure, and behavior. Therefore, it is important to complement laboratory studies with clinical observations to gain a comprehensive understanding of biofilm-associated infections in real-world medical scenarios [68].

The presence of biofilms on endotracheal tubes (ETTs) has significant implications for patient outcomes and healthcare management. These biofilms act as reservoirs for harmful microorganisms, increasing the risk of ventilator-associated pneumonia (VAP). Additionally, biofilms can obstruct ETTs, affecting ventilation efficiency and complicating airway management.

Biofilms are resistant to standard antimicrobial treatments, often requiring the replacement of contaminated ETTs, a procedure not without risks. Therefore, understanding biofilms’ formation, composition, and behavior on ETTs is crucial for developing effective prevention and treatment strategies. This knowledge can guide the development of new materials to combat biofilms, inform antimicrobial stewardship practices, and enhance patient outcomes in intensive care settings.

It is essential to prioritize preventing and treating biofilms to reduce the mortality and morbidity associated with VAP. Developing a quick bedside method for detecting bacteria within biofilms and finding ways to eliminate them could significantly improve the standard of care for critically ill patients. Preventive measures should also be taken to stop virulent bacteria from adhering to biotic and abiotic surfaces, particularly on medical devices.

The current methods for detecting biofilms on endotracheal tubes (ETTs) vary in their sensitivity and specificity, leading to inconsistent diagnoses of ventilator-associated pneumonia (VAP). Future research must concentrate on standardizing biofilm detection techniques and determining which methods are most predictive of infection in clinical settings. Biofilm formation on ETTs does not consistently indicate active infection, as bacterial colonization can occur without leading to VAP. Research is critical to developing improved markers or clinical criteria capable of differentiating between benign colonization and pathogenic biofilms that contribute to VAP. Ventilator-associated tracheobronchitis (VAT) is a condition in which biofilms are present without pneumonia and may progress to VAP. The correlation between VAT and VAP remains incompletely understood, warranting further studies to ascertain whether early treatment of VAT can forestall the development of VAP. Procedures such as suctioning and intubation can disrupt biofilms, potentially elevating the risk of VAP. Additional research is required to understand the effects of airway manipulations on biofilm dynamics and how this insight could shape preventive strategies [69].

## 5. Conclusions

This thorough review has highlighted the complex challenges presented by biofilm formation on endotracheal tubes, emphasizing the need to develop effective detection and characterization methods. By exploring various techniques for assessing biofilms, from microscopy to molecular diagnostics, this study has improved our understanding of the factors causing ventilator-associated pneumonia.

The analysis of different types of endotracheal tubes and the bacteria that live in the biofilms on these tubes provides important insights. These insights can help improve the design of antimicrobial strategies and medical devices. Ultimately, this research has the potential to improve clinical practices and patient outcomes by enabling earlier diagnosis, targeted interventions, and better infection control measures.

This study also emphasizes the urgent need for healthcare systems to work together to address biofilm-associated infections. It highlights the importance of translating research into practical improvements in patient care. By utilizing various research methods, clinicians and researchers can strive to prevent, reduce, and effectively manage complications such as ventilator-associated pneumonia. This joint effort is focused on protecting the health of vulnerable patient populations, like most of the patients admitted to ICUs.

## Figures and Tables

**Table 1:** Summary of the reviewed studies.

Authors	Biofilm Identification Method	Human	In Vitro	Animals	ETT	Microorganisms
Alves D. et al., 2023 [18]	Infrared spectroscopy		•		Double-coated ETT (ciprofloxacin and chlorhexidine)	*Ps. Aeruginosa*, *A. baumanii*, *K. pneumoniae*, *S. aureus*, *S. epidermidis*
Amar A.K. et al., 2022 [19]	16SrRNA gene amplification followed by Sanger sequencing; NGS of the device metagenome	•			Invasive medical devices	*S. infantis*, *Gemella haemolysans*, *Meiothermus silvanus*, *Schlegelella aquatica*, *Rothia mucilaginosa*, *Serratia nematodiphila*, *and Enterobacter asburiae*, along with some known common nosocomial pathogens
Azmi A. et al., 2023 [20]	Computed tomography		•		Laccase@GdPO4 * HNP, Enzyme mediator (antibiofilm property)	*E. coli* *S. aureus* *P. aeruginosa*
Bereanu A.S. et al., 2024 [21]	Not the case		•		Medical devices, including ETT–TiO_2_ photocatalytic	*K. pneumoniae*
Cifuentes E.A. et al., 2022 [22]	Sonication Powersoil Kit for DNA extraction	•			Usual ETT	*Proteobacteria* (*p* = 0.01), *Firmicutes*, *Bacteroidetes*, *Fusobacteria* (*p* = 0.01), *Actinobacteria* (*p* = 0.02)—bacteria species with statistical significance difference between the 2 ICUs Biofilm bacteria, patients with VAP *: *K. pneumoniae*, *E. coli*, *P. aeruginosa*, *A. baumanii*, *S. aureus*
Daengngam C. et al., 2019 [23]	Scanning electron microscopy	•			Coated ETT **	*S. aureus*, *P. aeruginosa*
Dewi F.H. et al., 2021 [24]	Quantitative biofilm measurement using a microtiter plate method, optical density	•			Usual PVC	48 specimens were obtained; Gram–negative bacteria were more common cause of VAP than Gram–positive bacteria (81% vs. 17%). There was one unidentified microorganism (2%)
Drago L. et al., 2024 [25]	Sonication DTT				Not the case	*A. baumanii*, *S. aureus*, *P. aeruginosa*, Enterobacteriaceae, *Citrobacter koseri*, *P. mirabilis*, *P. fluorescence*
Dsouza et al., 2019 [26]	Catheter-based OCT	•			Usual PVC	
Dsouza R. et al., 2021 [27]	OCT *	•			Usual PVC	*Klebsiella* spp. (mainly)
Fady M. et al., 2023 [28]	Scanning electron microscopy	•			Usual PVC	*S. aureus*, *S. epidermidis*, *P. vulgaris*, *K. pneumoniae*, and *P. aeruginosa*
Fernandez-Barat L. et al., 2017 [29]	Sonication			•	Not the case	*P. aeruginosa*, MRSA
Fernandez-Barat L. et al., 2019 [30]	Scanning electron microscopy	•			Usual PVC	MRSA
Fernandez-Barat L. et al., 2024 [31]	Real-time PCR for the assessment of genes in the biofilm			•	Usual PVC	MRSA
Gasparetto J. et al., 2022 [32]	No method for biofilm detection	•	•		Chlorhexidine-impregnated TST and violet-crystal-coated TST	Standard strains of *S. aureus*, *P. aeruginosa*, *E. coli*, and MDR bacteria (MRSA, carbapenem-resistant *A. baumannii*, *P. aeruginosa*, *K. pneumoniae*
Guilhen C. et al., 2019 [33]	Confocal Microscopy Sonication Flow cytometry		•	•	Not the case	*K. pneumoniae*
Jones C.J. et al., 2022 [34]	RNA-sequencing analysis		•		Usual PVC (and 2 other abiotic surfaces)	*P. aeruginosa*
Khazaal S.S. et al., 2020 [35]	No description of biofilm data collection	•			Usual PVC (bacteria was initially grown)	*A. baumannii*
Kiarostami K. et al., 2024 [36]	Scanning electron microscopy			•	Usual PVC	MRSA
Latorre M.C. et al., 2021 [37]	Cfu count by culture of sonicate and the total number of cells by confocal laser scanning microscopy		•		Ceragenin CSA-131 coated ETT and uncoated PVC ETT	*P. aeruginosa*, *S. aureus*, *E. coli*
Lethongkam S. et al., 2023 [38]	Energy dispersive X-ray spectroscopy			•	Eucalyptus-mediated synthesized silver nanoparticles (AgNPs)	*P. aeruginosa*
Luo Y. et al., 2021 [39]	Total internal reflection fluorescence microscopy	•			Usual PVC	*P. aeruginosa*, *E. coli*, *S. aureus*
Maldiney T. et al., 2022 [40]	Confocal microscopy MALDI-TOF MS *	•			Usual PVC	Mushroom-shaped BF *: *S. aureus*, *S. haemolitycus*, *S. epidermidis*, *E. coli*, *K. oxytoca*, *P. aeruginosa*, *Serratia marcescens*, *E. cloacae* (*p* = 0.002), *E. xiangfangensis* (*p* = 0.02), *E. faecalis*, *E. faecium*, *S. pneumoniae* (*p* = 0.009), *S. oralis* (*p* = 0.004), *Hafnia alvei* (*p* = 0.02) Ribbon-shaped BF *: *S. aureus*, *S. haemolitycus*, *S. epidermidis*, *E. coli*, *K. oxytoca*, *P. aeruginosa*, *Serratia marcescens*, *E. cloacae*, *E. faecalis*, *E. faecium*
Marcut L. et al., 2023 [9]	Scanning electron microscopy	•	•		Modified ETT ***	*E.* coli, *P.* aeruginosa, S. aureus (including MRSA)*,* and *B.* subtilis, K. pneumoniae, A. baumannii
Mazzolini R. et al., 2022 [41]	Crystal-violet assay, Alcian Blue	•	•	•	-	*P. aeruginosa*
Mishra S. et al., 2024 [42]	Multiple methods	•			Usual PVC	Specific bacteria from the EKAPE group
Oliveira V.C. et al., 2020 [43]	-		•		ETT with bacteriophages on the surface	*P. aeruginosa*
Oliveira V.C. et al., 2021 [44]	Sonication, Scanning Electron Microscopy		•		Phage cocktail adsorbed to ETT	*P. aeruginosa*
Ozcelik B. et al., 2020 [45]	No description		•		A novel styryl benzene-based antimicrobial (BCP3) coating	*S. aureus*, *P. aeruginosa*
Perez-Granda M.J. et al., 2020 [46]	Sonication Confocal laser scanning microscopy		•		Usual PVC	*P. aeruginosa*, *S. aureus*, *E. coli*
Rangel K. et al., 2024 [47]	Rapid molecular testing	•			Usual PVC	*A. baumannii*
Rao H. et al., 2021 [48]	-		•		Usual PVC (also venous catheter, urinary catheters)	ESKAPE group bacteria
Roy S. et al., 2022 [49]	Not the case	•			Usual PVC, coated ETT	*A. baumannii*
Shaqour B. et al., 2021 [50]	Scanning electron microscopy			•	TPU polymeric matrix with incorporated ciprofloxacin	*S. aureus*
Soares R.B. et al., 2020 [51]	Crystal violet absorbance		•		Methylene blue associated with external illumination	*P. aeruginosa*
Thorarinsdottir H.R. et al., 2020 [5]	Electron microscopy	•			Uncoated PVC Silicon-coated PVC PVC coated with noble metals	*E. faecalis*, *E. faecium*, *S. aureus*, *Klebsiella* spp., *Stenotrophomonas maltophilia*, *P. aeruginosa*
van Charante F. et al., 2022 [52]	Culture-dependent (MALDI-TOF mass spectrometry and biochemical tests) and culture-independent (16S and ITS1 rRNA amplicon sequencing)	•			Usual PVC	*S. epidermidis*, *E. faecalis*, *P. aeruginosa*
Walsh D. et al., 2024 [53]	Matrix-degrading enzymes and cryo-SEM		•		PVC ETT segments in the presence of synthetic ventilator airway mucus	*P. aeruginosa*, *K. pneumoniae*
Zangirolami A.C. et al., 2020 [54]	FT-IR* spectroscopy		•		PVC coated with curcumin-photosensitizer	*P. aeruginosa*, *S. aureus*, *E. coli*

* Biofilm; ** Polyelectrolyte multilayered film that accommodated silver nanoparticles (AgNPs) formed in situ by reducing Ag^+^ ions with Eucalyptus citriodora leaf extract; *** Noble metal inserted in or coated on polymeric surfaces. Some metallic materials or their oxides, such as silver (Ag), selenium (Se), silver oxide (Ag_2_O), titanium dioxide (TiO_2_), iron oxides (Fe_2_O_3_, Fe_3_O_4_), zinc oxide (ZnO), and copper oxide (CuO). Bio-Inspired Antimicrobial Coatings: Antimicrobial peptides (AMPs), bacteriophage coatings, and coatings based on surfactant (including carrageenin). Nanomodified hydrophilic/hydrophobic surfaces. Micropatterning surface modification. Combination materials.

## Data Availability

The data are contained within this article.

## Abbreviations

BF	biofilm
CAMPs	cationic antimicrobial peptides
CIP/CHX	Ciprofloxacin/Chlorhexidine
Cryo-SEM	cryo-scanning electron microscopy
DRI	device-related infections
DTT	dithiothetriol
EPS	extracellular polymeric substances
ESKAPE	*Enterococcus faecium*, *Staphylococcus aureus*, *Klebsiella pneumoniae*, *Acinetobacter baumannii*, *Pseudomonas aeruginosa*, *Enterobacter* species
ETT	endotracheal tube
FT-IR	Fourier Transform InfraRed
ICU	intensive care unit
OCT	optical coherence tomography
TiO_2_	titanium dioxide
TST	tracheostomy tube
VAP	ventilator-associated pneumonia
